# Identifying association under a previous linkage peak on chromosome 16 for body mass index using cross-sectional and longitudinal data of the Framingham Heart Study

**DOI:** 10.1186/1753-6561-3-s7-s101

**Published:** 2009-12-15

**Authors:** Xiaohui Li, Ling Mei, Kai Yang, Jerome I Rotter, Xiuqing Guo

**Affiliations:** 1 Medical Genetics Institute, Cedars-Sinai Medical Center, 8635 West Third Street, Suite 1150, Los Angeles, California 90048 USA

## Abstract

We performed association analysis under a previous linkage peak on chromosome 16 with genome-wide single-nucleotide polymorphism (SNP) data to identify genetic variants underlying body mass index (BMI). Data from all subjects with baseline measures and a subgroup who had complete data at four selected time points from the Framingham Heart Study were analyzed. The cross-sectional measures include BMI at baseline for all subjects, as well as BMI at selected time points for the subgroup. The longitudinal measure is the within-subject mean of BMI for the subgroup at the four time points.

Association analysis was first performed using PLINK after dividing large pedigrees into nuclear families. We then followed up the identified regions by variance-components methods as implemented in SOLAR using the extended pedigrees.

The strongest evidence for associations were observed at 52.3 Mbp (PLINK *p *= 0.00002, QTLD *p *= 0.005), on the *FTO *gene, and at 48.1 Mbp (PLINK *p *= 0.002, QTLD *p *= 0.0006) on chromosome 16, which are directly under the previous identified linkage peak. This association was consistently observed for all samples at baseline, and for the subgroup at time point 2, 3, 4 and MEAN, both by PLINK and SOLAR. In addition, another SNP/region at 46.7 Mbp on same chromosome was found to be associated with several BMI measures in the subgroup. Fine-mapping with more markers provided further evidence for SNP association with BMI in the same region (at 52.4 Mbp, QTLD *p *= 0.0003).

These results suggest the existence of genes/DNA variations in these regions that contribute to BMI variation.

## Background

Obesity, which is influenced by both genetic and environmental factors, is an independent risk factor for coronary heart disease (CHD). However, the reported quantitative trait loci (QTLs) for body mass index (BMI) are not consistent. The discrepancy among various studies represents the difficulty in identifying susceptibility genes for common complex traits. Many factors, including, but not limited to sample size, ascertainment method, long-term or short-term environmental influences, and genetic heterogeneity, all may contribute to the discrepancy among studies. Age-dependent penetrance of a trait is another factor that may complicate the identification of susceptibility genes.

Cross-sectional study designs collecting phenotypic data at one time point are used in most genetic studies, mainly due to their relative feasibility. However, longitudinal data may answer questions that cross-sectional data cannot. A longitudinal study with measures at multiple time points might be the best solution for gene mapping studies of traits with age-dependent penetrance. Furthermore, serial observations over time of the same trait may allow more accurate partitioning of genetic and environmental components than would a single observation [1]. Thus, longitudinal data may provide more and/or different insight in dissecting the genetics of a complex trait.

The Framingham Heart Study (FHS) is a successful longitudinal study of cardiovascular diseases. Started in 1948, FHS has obtained longitudinal cardiovascular-related phenotypic measures in two generations of participants. A 10-cM genome-wide scan in subjects from 330 families was carried out in the late 1990s and several suggestive linkage regions were identified for BMI [2]. The highest linkage peak was observed on chromosome 16 and it was consistent for each time point. Lately, 500 k single-nucleotide polymorphisms (SNPs) across the genome and 50 k SNPs in follow-up candidate genes were genotyped. This provided a unique opportunity to map QTLs for CHD-related traits.

Our goals in this study were: 1) to identify QTLs for BMI using an association approach, taking into account the linkage information we had obtained in the previous genome-wide linkage studies [2]; and 2) to assess the consistency of QTLs identified over time using the FHS data. Follow-up association tests for SNPs within the previously identified linkage regions and evaluation of association with multiple time point measures should increase the power to identify the true positives. In order to improve the power of identifying true-positive association (i.e., considering linkage information), and avoid a huge number of multiple tests, we focused our association study on chromosome 16.

## Methods

### Subjects

Three cohorts were recruited in the FHS. Both the Original Cohort (Generation 1) and Offspring Cohort (Generation 2) had data collected at baseline and three follow-up visits. The Generation 3 had only baseline measures. Using the cross-sectional measures from baseline, a total of 960 extended pedigrees consisting of 6475 subjects were used in the genome-wide association analysis of BMI.

In order to evaluate the longitudinal effect and to make association analysis results from different time points comparable, we also analyzed those subjects with phenotypic data at all the four selected visits as a subgroup (Original and Offspring Cohorts only). Only families with at least two blood-related individuals who met such criteria were included in the analysis.

### Phenotype

BMI [weight (kg)/height (m)^2^] was selected as the quantitative trait because of its completeness and uniform method of measurement over time. Baseline BMI was denoted as BMI-B. For the subgroup of the Original and Offspring Cohorts, five measures of BMI, denoted as BMI1, BMI2, BMI3 and BMI4 (Visit 1, 4, 7, 11 for the Original Cohort; Visit 1, 3, 5, 7 for the Offspring Cohort) as well as MEAN (the within-subject mean of the four visits over years), were analyzed.

### Statistical analysis

Data quality control (QC) was performed using PLINK to remove SNPs with minor-allele frequency (MAF) less than 0.05 and to zero out mendelian inconsistencies [3]. SNPs with higher missing rate (>0.1) or failing the Hardy-Weinberg test at 0.01 significance level were also excluded from analysis. Samples with lower genotyping rates (<0.9) were removed. Approximately two-thirds of SNPs passed QC and were used in the association analysis. In addition, Relcheck was also performed to identify errors in the familial relationships [4]. Problematic individuals were excluded for further analysis.

Because of the limitations of the analysis program and time constraints, the association between each SNP and BMI was first evaluated using nuclear families (by dividing the large pedigrees) and the program PLINK [3] with 100,000 permutations. PLINK assesses association by the linear model and adjusts for family relatedness by permutation. Because the total number of SNPs on chromosome 16 was around 10,800, we set the criteria for significant SNP selection as *p *< 0.0001 and being located under the identified linkage peaks.

Follow-up association analysis for the selected region and fine-mapping were conducted using extended families by the variance-components analysis method as implemented in the SOLAR program [5,6]. Three methods were utilized for association test by SOLAR: measured genotype analysis, quantitative trait transmission disequilibrium test (QTDT) and quantitative trait linkage disequilibrium (QTLD). The orthogonal model was used in the QTDT method, in which the total association is partitioned into orthogonal within- and between-family components. The between-family component was sensitive to population stratification, while the within-family structure was free of confounding by family structure effect and was significant only in the presence of linkage disequilibrium. QTLD was a modification of QTDT, which considered the founders' information when population stratification is absent [6].

Association test for quantitative traits was performed on BMI-B, BMI1-BMI4, and the MEAN under the additive model. Age, sex, and cohort were included as covariates in the association analyses.

## Results

### Association for baseline BMI (BMI-B) using genome-wide association study (GWAS) data on chromosomes 16

**Figure 1:**
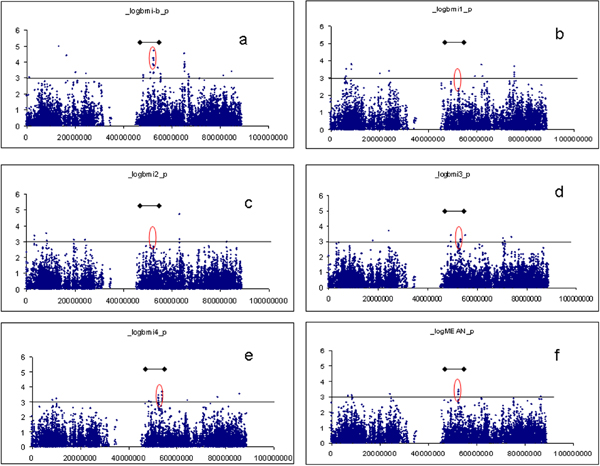
**Association test of BMI-B, BMI1, BMI2, BMI3, BMI4, and MEAN on chromosome 16 by PLINK**. a, BMI-B; b, BMI1; c, BMI2; d, BMI3; e, BMI4; f, MEAN. Black line with dots: 10-cM range of linkage peak.

Using divided nuclear families (*n *= 6475 subjects), 5 SNPs (52.3 Mbp) met the selection criteria, i.e., *p *< 0.0001 and located under the previous identified linkage region (highest LOD = 3.0 at 45 cM) [2] on chromosome 16. Several other SNPs had *p *= 0.001 and were within 10 cM of the peak (45-55 Mbp) (Figure 1).

Applying SOLAR to extended families, the top SNP was found to be rs11647781 at 48.1 Mbp (PLINK *p *= 0.002, QTLD *p *= 0.0006), while the SNPs identified by PLINK at 52.3 Mbp remained significant. The top SNP in the latter region was rs9926289 (PLINK *p *= 0.00002, QTLD *p *= 0.005, Table 1). In general, *p*-values were consistent from the three different methods (measured genotype analysis, QTDT, and QTLD) and so we only reported the *p*-value from QTLD in Table 1.

**Table 1:** *p*-Value for associated SNPs under the linkage peak (*p *< 0.0001 by PLINK or *p *< 0.001 by QTLD for one or more BMI measures)

SNP	Position (bp)	BMI-B		BMI1		BMI2		BMI3		BMI4		MEAN	
							
		PLINK	QTLD	PLINK	QTLD	PLINK	QTLD	PLINK	QTLD	PLINK	QTLD	PLINK	QTLD
**rs16945869^a^**	**46732060**	**0.219**	**0.026**	**0.004**	**7.8 × 10^-6^**	**0.006**	**0.0002**	**0.039**	**0.0006**	**0.001**	**10^-5^**	**0.002**	**4.6 × 10^-6^**
**rs11647781**	**48151920**	**0.002**	**0.0006**	**0.024**	**0.04**	**0.023**	0.020	0.030	0.31	**0.005**	**0.021**	**0.011**	**0.024**
rs4785326	48173088	0.014	0.001	0.06	0.07	0.15	0.034	0.074	0.22	0.041	0.09	0.048	0.05
rs9939973	52358069	0.00014	0.010	0.019	0.18	0.002	0.06	0.001	0.016	0.00061	0.028	0.00044	0.012
rs9940128	52358255	0.00006	0.008	0.022	0.22	0.003	0.08	0.002	0.023	0.00051	0.028	0.00048	0.017
rs1121980	52366748	0.00016	0.011	0.023	0.22	0.002	0.09	0.002	0.025	0.00037	0.027	0.00035	0.017
rs7193144	52368187	0.00008	0.006	0.019	0.12	0.003	0.07	0.003	0.007	0.001	0.011	0.00074	0.006
rs8050136	52373776	0.00006	0.007	0.023	0.15	0.004	0.10	0.003	0.008	0.001	0.009	0.00069	0.007
**rs9926289**	**52378004**	**0.00002**	**0.005**	**0.020**	**0.25**	**0.003**	**0.25**	**0.004**	**0.030**	**0.00086**	**0.023**	**0.00077**	**0.029**
rs9939609	52378028	0.00002	0.008	0.018	0.15	0.003	0.12	0.002	0.010	0.001	0.013	0.00049	0.008

^a^Bold font indicates the most significant SNPs.

### Association for four time points (BMI1, BMI2, BMI3 and BMI4) and MEAN BMI for the subgroup using GWAs data

Although using a subgroup of samples (*n *= 2702) may reduce the power to identify the association, we observed SNP associations similar to BMI-B for BMI2, BMI3, BMI4, and MEAN (Table 1). Interestingly, an additional SNP (rs16945869, 46.7 Mbp) was identified to be associated with all five BMI measures in this subgroup.

### Fine-mapping on selected regions

Within the same region, we selected 150 additional SNPs from the fine-mapping markers to further test the associations for all subjects and the subgroup samples. Only one fine-mapping SNP in the same region (rs2042032, 52.4 Mbp) showed significant association (QTLD *p *= 0.0004) for baseline BMI for all subjects. Another fine-mapping SNP (rs16945874, 47.6 Mbp), which is in the same region as the SNP identified in the subgroup samples (rs16845869), also remained significant (QTLD *p *= 2 × 10^-6 ^for BMI1 and MEAN) in this subgroup.

## Discussion

We performed association analyses on chromosome 16 using genome-wide scan SNP data for BMI measured at different time points and the mean of BMI over a number of years on the FHS sample. The strongest evidence for association was observed at 52.3 Mbp and 48.1 Mbp, which are directly under the previously identified linkage peak. The most consistent associations were observed in baseline BMI for all samples and most time points and MEAN for the subgroup by different analysis methods. The *p*-values tended to be smaller for all samples, possibly due to the relatively larger sample size. Fine-mapping with more markers confirmed the associations in the same region. Two additional SNPs at 46.7 Mbp (one from the original GWAS and the other from fine-mapping) were identified in the subgroup only.

One of the major issues for GWAS is separating true and false positives due to the huge number of multiple tests. Combining the information from previous linkage studies may provide additional power to identify true associations. On chromosome 16, the second most significant *p*-value and several tentative associations was observed under the previous identified linkage region, and the evidence for association were consistently observed by different statistical analysis methods as well as for different samples. Considered these together, we believe that there must be some genes or DNA variants in this region that contribute to the determination of BMI. The most significant region we identified (52.3 Mbp) harbors *FTO*, a gene which was found to be associated with fat mass and obesity. Recent studies have reported that the variants of this gene are associated with body fat distribution and a variety of metabolic traits, such as insulin resistance, diabetes, and high-density lipoprotein cholesterol level [7].

Using longitudinal observations of BMI, we derived MEAN as a longitudinal phenotype that represents an overall status of BMI over years. Although the sample size was much smaller (2702 vs. 6475), we identified similar SNPs as those found in the analysis of all samples at baseline only. In general, BMI4 (at time point 4) and MEAN yield similar associations. Even though it is expected that longitudinal measures provide more information, this was not proven in the study. One possible explanation is the much-reduced sample size when using the longitudinal data.

It is intriguing that two top hits (one from fine-mapping) were identified in the subgroup only. After adding more samples from Generation 3 (mean age at baseline was 40.1 compared with 33.8 for the subgroup), the association in the subgroup at 47.6 Mbp was diluted. It is noteworthy that the three different generations in the FHS may have different environmental exposures. Thus, unmeasured environmental factors that may interact with genes might be a major issue in genetic studies.

A limitation of our study is that we only performed univariate analyses, which may not be able to identify causative genes or SNPs that are individually weak but collectively informative. Multivariate approaches will be helpful in identifying those SNPs. As far as we know, however, the available machine-learning methods were developed for random individuals or case-control data, which restricts their applications to the FHS data, which contained extended pedigrees.

## Conclusion

In summary, there appears to be genetic variation(s) on chromosome 16 that contribute to BMI. In addition to age, environmental factors, and major genes with marginal effects, there may also be different genes that determine BMI variations at different age periods. Nevertheless, when we analyzed cross-sectional BMI data at different time points and the mean for all time points, we observed consistent associations with BMI at 52.3 Mbp, i.e., the *FTO *gene. Another region (47.6 Mbp) may contain genes contributing to BMI by interacting with environmental factors.

## List of abbreviations used

BMI: Body mass index; BMI-B: Baseline BMI; CHD: Coronary heart disease; FHS: Framingham Heart Study; GWAS: Genome-wide association study; MAF: Minor-allele frequency; QC: Quality control; QTDT: Quantitative trait transmission disequilibrium; QTL: Quantitative trait locus; QTLD: Quantitative trait linkage disequilibrium; SNP: Single-nucleotide polymorphism.

## Competing interests

The authors declare that they have no competing interests.

## Authors' contributions

XL participated in the design and analysis and drafted the manuscript. LM and KY participated in data cleaning and analysis. JIR helped to draft the manuscript. XG participated in coordination and helped to draft the manuscript. All authors read and approved the final manuscript.
